# The Use of the Target Cancellation Task to Identify Eloquent Visuospatial Regions in Awake Craniotomies: Technical Note

**DOI:** 10.7759/cureus.883

**Published:** 2016-11-17

**Authors:** Andrew K Conner, Chad Glenn, Joshua D Burks, Tressie McCoy, Phillip A Bonney, Ahmed A Chema, Justin L Case, Scott Brunner, Cordell Baker, Michael Sughrue

**Affiliations:** 1 Department of Neurosurgery, University of Oklahoma Health Sciences Center; 2 Department of Physical Therapy, University of Oklahoma Health Sciences Center; 3 Department of Neurological Surgery, University of Southern California

**Keywords:** target cancellation, line bisection, awake craniotomies, mapping, neglect

## Abstract

The success of awake craniotomies relies on the patient’s performance of function-specific tasks that are simple, quick, and reproducible. Intraoperative identification of visuospatial function through cortical and subcortical mapping has utilized a variety of intraoperative tests, each with its own benefits and drawbacks. In light of this, we developed a simple software program that aids in preventing neglect by simulating a target-cancellation task on a portable electronic device. In this report, we describe the interactive target cancellation task and have reviewed seven consecutive patients who underwent awake craniotomy for parietal and/or posterior temporal infiltrating brain tumors of the non-dominant hemisphere. Each of these patients performed target cancellation and line bisection tasks intraoperatively. The outcomes of each patient and testing scenario are described. Positive intraoperative cortical and subcortical sites involved with visuospatial processing were identified in three of the seven patients using the target cancellation and confirmed utilizing the line-bisection task. No identification of visuospatial function was accomplished utilizing the line-bisection task alone. Complete visuospatial function mapping was completed in less than 10 minutes in all patients. No patients had preoperative or postoperative hemineglect. Our findings highlight the feasibility of the target cancellation technique for use during awake craniotomy to aid in avoiding postoperative hemineglect. Target cancellation may offer an alternative method of cortical and subcortical visuospatial mapping in patients unable to perform other commonly used modalities.

## Introduction

Anatomo-functional correlates have allowed surgeons to perform surgery with awake cortical and subcortical brain mapping to preserve neurologic function [1]. The validity and usefulness of awake craniotomy are dependent upon intraoperative testing with tasks that are reproducible, simple, and rapid for patients to complete.

Although there is no gold standard for visuospatial function mapping, the line bisection test has been commonly employed at our institution [2]. The line bisection test is an important tool in preventing postoperative hemineglect through the identification and, thus, avoidance of positive visuospatial sites [3]. However, this task relies on the observer‘s (surgeon) interpretation of subtle shifts from the midline, thus, losing some objectivity in real-time data analysis and potentially failing to identify early changes in function. Given this challenge, we developed a simple software program that aids in preventing neglect by simulating a target-cancellation task on a portable electronic device.

Here, we describe the feasibility of the target cancellation task for use during awake craniotomy, as well as compare the target cancellation task to the line bisection task in a small group of patients. We review the technique and rationale for target cancellation, discuss an illustrative case, and report early postoperative outcomes in regards to hemineglect.

## Technical report

Preoperative evaluation

Between January and September of 2015, six consecutive patients underwent awake craniotomy and performed the target cancellation task as well as the line bisection task intraoperatively; the seventh patient was unable to perform the line bisection task due to fine motor limitations but was able to successfully complete the target cancellation task (Table 1). Approval for this study was obtained from the University of Oklahoma Health Sciences Center Institutional Review Board, approval #3199. Informed patient consent was obtained. All patients harbored infiltrative gliomas either in the parietal lobe (1/7; 14%) or posterior temporal lobe (6/7; 86%). Lesions were pathologically graded according to the World Health Organization guidelines. Preoperative magnetic resonance imaging included image guidance sequences and diffusion tensor imaging (DTI) fiber tractography [4]. Preoperatively, all patients underwent a complete neurological examination and were evaluated by ancillary support specialists from physical therapy and speech-language pathology. In every instance of preoperative preparation for the awake craniotomy, the tasks that were to be utilized in surgery were tested and confirmed with each patient. This allowed us to have a preoperative baseline.

**Table 1:** Patient Characteristics GBM: glioblastoma multiforme; AA: anaplastic astrocytoma

Patient No.	Age / Gender	Pathology	Location
1	68 / M	GBM	Posterior temporal
2	56 / M	AA	Posterior temporal
3	59 / F	GBM	Posterior temporal
4	59 / M	GBM	Posterior temporal
5	56 / M	GBM	Posterior temporal
6	48 / F	GBM	Posterior temporal
7	52 / M	GBM	Inferior parietal

At our institution, the intraoperative mapping protocol is tailored to lesion location but generally includes functional tasks, such as counting, naming, reading, motor planning and function, and assessment of neglect. After training, patients were instructed to complete line bisection and target cancellation tasks to assess visuospatial function. Intraoperative cortical and subcortical mapping of visuospatial function was performed with the use of the target cancellation task and the line bisection test.

The rationale for performing awake craniotomies in these patients was centered on the proximity to eloquent regions as assessed by preoperative MRI and tractography. In some patients, the tractography did not identify all relevant major white matter fibers. This may occur in the setting of tumor infiltration or cerebral edema. Because preoperative testing failed to identify deficits that would be anticipated with disruption of the relevant major white matter fibers, these networks were assumed to be intact. With their location not estimated by tractography, cortical and subcortical mapping was felt to be the safest option in order to avoid a postoperative deficit.

Mapping technique

As keyhole craniotomies are designed to expose the minimum amount of cortical surface necessary to safely resect a tumor, we performed negative cortical and subcortical mapping in order to define functional resection boundaries in all patients [5]. Craniotomies are planned based on tumor/eloquent cortex boundaries with the intent to maximally resect the tumor burden. This is substantially aided by superimposed intraoperative DTI fiber tractography, which, in our experience, aids in the identification of eloquent cortical and subcortical regions and, thus, operative planning [2]. Stimulation was carried out at the lowest current that produced a deficit or did not result in after-discharges. Anatomical sites were stimulated a minimum of three times. Reproducibility was defined as three consecutive responsive stimulations.

Patients are asked to perform any task to be tested during mapping at the initial examination. It is then determined whether or not a patient is able to perform the intended tasks at neurologic baseline. The preoperative function can vary widely depending on pathology and lesion location [6]. Accurate assessment and determination of preoperative neurologic function allow for proper selection of intraoperative tasks. Hence, severe baseline deficits of a particular function (i.e., when the patient cannot complete the intended task) indicate that testing of this system is unlikely to provide meaningful information during surgery.

Target cancellation task

**Figure 1:**
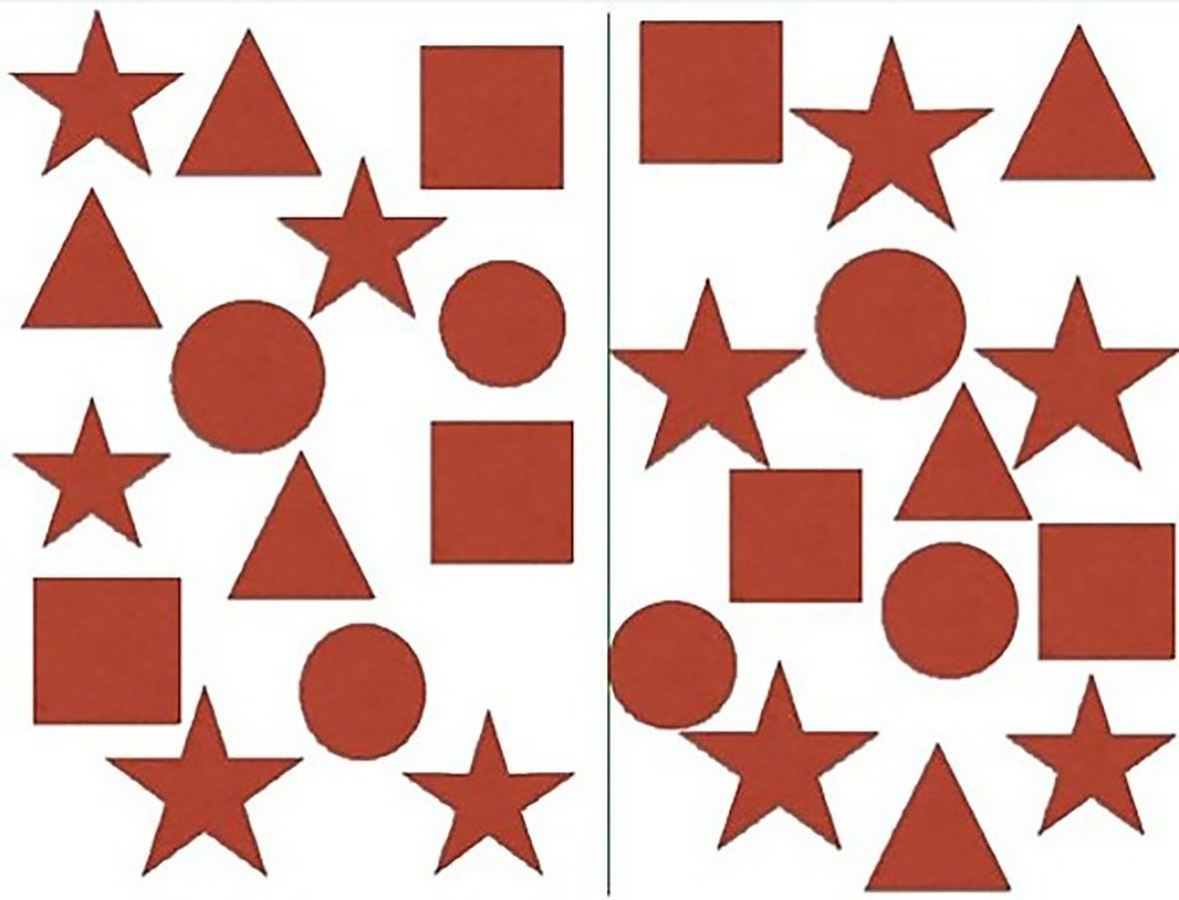
Target Cancellation Task An array of shapes is presented to the patient on a tablet. The shapes are equally allotted to each side of the screen. The patient is instructed to cancel the specified targets by swiping them with his or her finger.

In the target cancellation task, patients are shown an array of simple but distinct shapes that are placed at random with a bisecting vertical line dividing the array into equal halves (Figure 1). The array is presented on a handheld tablet computer with touchscreen capabilities utilizing a simple program developed at our institution. Patients are instructed to mark or “cancel” the specified targets or objects in both the right and left fields. The patient will then place his or her finger onto the tablet screen overlying the specified shape and effectively cancel the shape by making a swiping movement (Video 1). Stimulation is continuous as the patient cancels each object in the array.

**Video 1:** Target Cancellation Task

Upon completion of this task, the ratio of successfully canceled objects on each side of the object array is rapidly determined. For example, the patient may be asked to cancel all of the “square” shapes as seen. With an equal number of “square” shapes on each side of the array, an objective, quantitative interpretation can be made. As the surgeon stimulates various cortical and subcortical sites, different cancellation arrays are used. Early signs of neglect become apparent when the patient is unable to select the appropriate objects on one side of the array, as an independent concentration with each cerebral hemisphere is necessary to perform the tasks across the midline. During cortical stimulation, a diminished score on one side compared to the other, or compared to baseline function, may, therefore, indicate proximity to structures involved in visuospatial function, thus, generating a positive site. In general, patients who are able to perform this task at baseline do so with a high level of reproducibility, making diminishing scores readily noticeable. For the sake of our study, a site was considered to map positive if the patient failed to cancel one or more objects in target cancellation with lateralized predominance. Total stimulation time for most patients to readily complete the task is approximately 20-30 seconds. The completion of each task is signaled by the patient’s verbal confirmation that all shapes have been identified/canceled.

Line bisection task

In the line bisection task, patients are shown a line stretching across the tablet screen and are instructed to “bisect the line,” by drawing a mark in the center of the image. Lateral deviation of the patient’s mark from the midline of approximately 5 mm is considered a positive result, thus, correlating with a functionally significant visuospatial region. Again, total stimulation time for most patients to readily complete the task is approximately 20-30 seconds. The completion of each task is signaled by the patient’s verbal confirmation that the task is complete.

Intraoperative nuances

Care was taken to maintain each visual field within the corresponding half of the object array, avoiding visual field crossover and resultant inaccurate cancellation results. Placing the tablet directly at the patient’s midline, and in some cases, securing a small divider to the bridge of the patient’s nose or glasses, blocked visual field crossover.

Postoperative evaluation

Postoperatively, all patients underwent a complete neurological examination and were evaluated by ancillary support specialists from physical therapy and speech-language pathology. Comparison of task outcomes both pre- and postoperatively were recorded. 

Operative results

The target cancellation task was performed in all seven patients and the line bisection task in six patients undergoing awake cortical and subcortical mapping craniotomies for gliomas in the nondominant parietal and posterior temporal lobes for identification of regions involved with visuospatial function (Table 1). No patients had preoperative hemineglect.

Structures involved with visuospatial processing were positively mapped in three of the seven patients with the target cancellation task. In two of those three patients, sites that were positively mapped using the cancellation task were also positively mapped with the line bisection task. The other patient who mapped positive with target cancellation was unable to perform the line bisection test due to fine motor limitations. Table 2 shows target cancellation and line bisection results for all patients. Sites mapping positively were preserved during tumor resection.

**Table 2:** Evaluation with Target Cancellation ┼ Number of tasks successfully performed in the preoperative evaluation ╬ Number of tasks not performed successfully during stimulation. Resection was halted with positive mapping. A site was considered to map positive if the patient failed to cancel one or more objects in target cancellation or if line bisection deviated by more than 5 mm. *Patient was unable to perform line bisection intraoperatively due to fine-motor limitations TC: target cancellation; LB: line bisection

Patient No.	Test	Preop Tasks^┼^	Positive Mapping Sites^╬^	Postop Neglect
1	TC LB	3/3 3/3	0/5 0/3	No
2	TC LB	3/3 3/3	0/8 0/7	No
3	TC LB	3/3 1/3	3/5 2/4	No
4	TC LB	3/3 3/3	0/15 0/7	No
5	TC LB	3/3 3/3	5/5 3/3	No
6	TC LB	3/3 3/3	0/12 0/6	No
7*	TC LB	3/3 3/3	1/5 -	No

Complete target cancellation mapping included multiple object arrays and was completed in less than 10 minutes in all patients, with each array requiring less than 9 seconds. No seizures occurred during testing. The postoperative evaluation revealed no evidence of neglect in any patient.

Illustrative case

**Figure 2:**
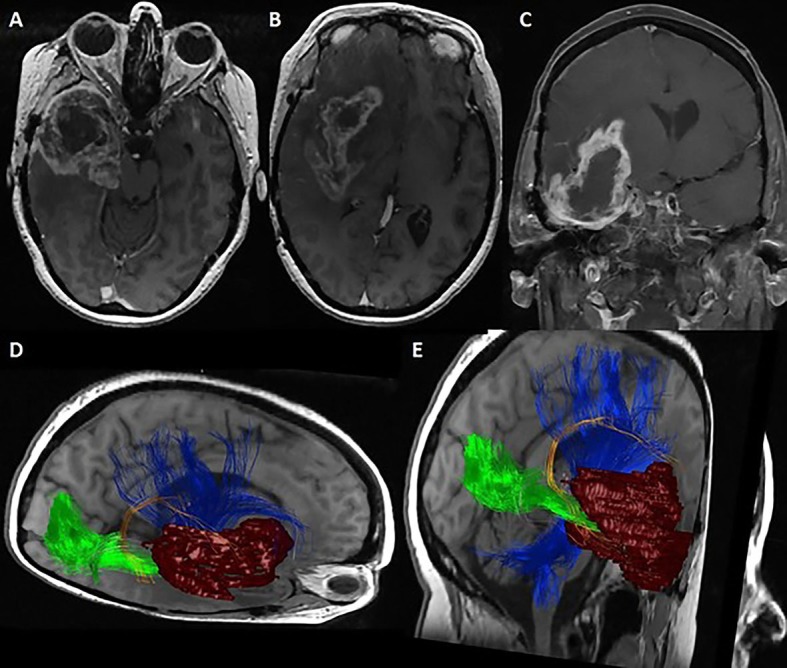
Illustrative Case Axial (A-B) and coronal (C) T1-weighted contrast-enhanced MRI demonstrates a heterogeneously enhancing mass located within the right frontal lobe, temporal lobe, and insula. (D-E) Diffusion tensor imaging (DTI) fiber tractography overlying reconstructed T2-weighted images in combined (D) axial-sagittal and (E) coronal-sagittal planes. The corticospinal tract is outlined in blue, optic radiations in green, superior longitudinal fasciculus in orange, and inferior frontoccipital fasciculus in pink. The inferior frontal occipital fasciculus (IFOF) is scarcely visualized due to tumor infiltration or compression. A tumor model outlining the enhancing portions of the tumor has been constructed in maroon for ease in visualizing its relationship to the relevant white matter tracts.

Figure 2 reveals the preoperative MRI and DTI tractography in a patient with recurrent glioblastoma of the right temporal lobe and insula who presented with worsening seizures and left extremity weakness.

**Figure 3:**
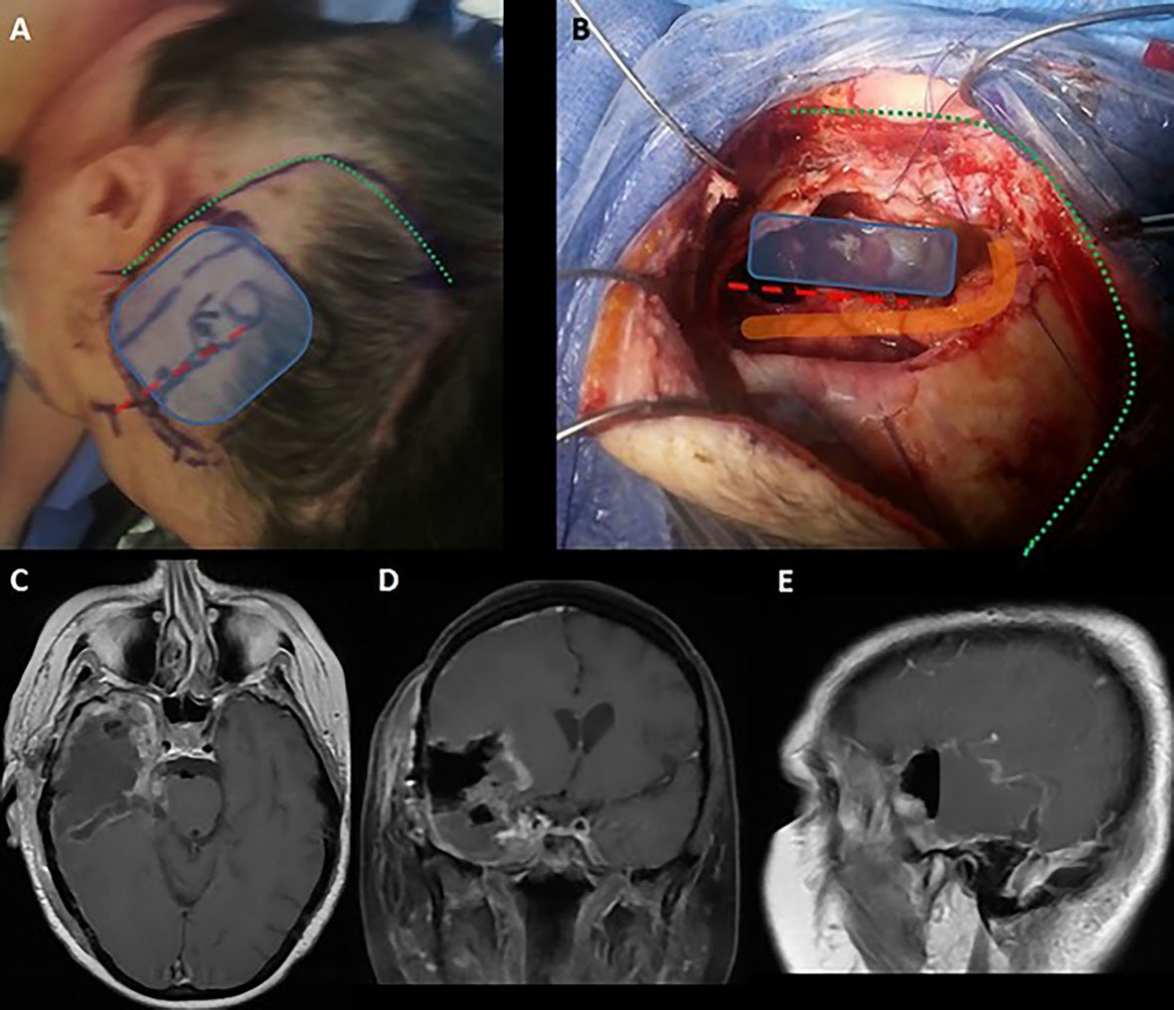
Procedure Description (A) Planned incision along previous craniotomy incision (green dotted line). Frameless navigation was used to identify the approximate area of tumor at cortical surface (blue box). The Sylvian fissure is marked with a red dashed line. (B) Intraoperative photograph revealing resection cavity. Cortical stimulation of the area outlined in orange induced reduced target cancellation scores and line deviation to the right. These cortical areas were not resected out of concerns for development of postoperative neglect. (C-E) Postoperative T1-weighted contrast-enhanced MRI images of (C) axial, (D) coronal, and (E) sagittal planes demonstrating tumor resection.

The enhancing portion of the tumor is visualized in dark red in DTI, revealing the proximity to the superior longitudinal fasciculus (SLF), inferior frontal occipital fasciculus (IFOF), optic radiations, and corticospinal tract (corresponding colors are given in Figure 2 legend). The IFOF is minimally visualized, likely due to tumor infiltration or compression. The preoperative neuropsychological evaluation demonstrated perfect scores on the target cancellation task and line bisection without deviation from the midline. During mapping at the posterior-superior margin of the temporal lobe, intraoperative cortical stimulation of the area outlined in orange (Figure 3) resulted in reduced target cancellation on the left field. This corresponded to a right deviation on line bisection. Further manipulation of these cortical sites was avoided. Subcortical stimulation did not identify eloquent structures within the margins of the resection cavity. Postoperatively, the patient demonstrated mild improvement of left extremity weakness and displayed no evidence of neglect.

## Discussion

Mapping motor and language areas have become increasingly common in the resection of tumors from eloquent regions [7], and efforts are underway to identify practical methods for mapping higher cortical functions [8-11]. The anatomic areas mapped during the assessment of visuospatial function and attention include the parietal lobe, the posterior-superior temporal lobe near the supramarginal and angular gyrus, as well as associated subcortical tracts, including the SLF and IFOF. Eloquent cortical and subcortical structures involved in visuospatial function exhibit marked variability between patients [10]. For this reason, cortical and subcortical mapping with the aid of diffusion tensor imaging tractography is employed in an effort to maximize the extent of resection while preserving function.

In this study, we introduce the target cancellation technique for identifying/defining the visuospatial network in awake craniotomies. We report seven cases of parietal or posterior temporal lobe gliomas on the non-dominant side in which both the target cancellation task and the line bisection task were employed during negative cortical and subcortical mapping. Three of these patients mapped positive for neglect with target cancellation with two out of three patients also mapping positive for neglect in regards to the line bisection task. Postoperatively, the visuospatial function was preserved in all patients studied.

The inferior and superior parietal, angular, and supramarginal gyrus, along with the non-dominant SLF, have been investigated with intraoperative mapping, given their hypothesized relationship to visuospatial and attention functions [2, 12]. Mapping to define eloquence in regards to visuospatial function has traditionally been performed with the line bisection test to prevent postoperative neglect [10]. A major limitation of this technique lies in some patients’ inability to participate intraoperatively. The line bisection test relies on the observer’s interpretation of a deviation from the midline and, thus, can underestimate early signs of neglect.

The target cancellation task involves distinguishing preselected objects in an array of distracting objects separated by a bisecting line. It can be performed on a small, portable electronic device and is easy for patients to complete in most circumstances. During the task, the preselected objects are identified or “canceled.” Correctly canceled objects are compared between respective visual fields; this generates a ratio between the numbers of appropriately canceled objects to the total number of intended cancellation targets in each half of the array (see intraoperative video). In this sense, target cancellation presents a clean, quantitative result that is easily and rapidly interpreted. The target cancellation task may be completed with a new object array immediately available for repeated testing during subcortical dissection or confirmatory cortical mapping.

It is possible that the target cancellation task and the line bisection task may investigate two distinct regions of the visuospatial network. Earlier authors have suggested that, in non-dominant hemineglect, the patient bisects the horizontal line to the right of center because he or she perceives the left half of the line to be shorter than the right half [13]. Conversely, in target cancellation, the patient cancels fewer targets on the left because of an attention deficit on the left. Yet, as each test likely utilizes distinct circuitry, both appear sensitive for detection of areas affected in neglect. Recently, in a moderately sized study evaluating hemineglect in stroke patients, target cancellation has been demonstrated to be superior to line bisection in regards to sensitivity when evaluating for hemineglect [14]. In this small series of patients, no positive areas of neglect were identified with only line bisection testing.

As with any test administered during awake mapping, target cancellation is potentially subject to misinterpretation or subversion. If the patient is able to look across the midline, he or she may be able to cancel targets that would not have otherwise been present in a neglected field. This issue may be addressed with a visual field divider when necessary.

Because target cancellation involves the interpretation of visual information combined with motor function, it is possible that disruption of the task may be caused by stimulation of eloquent areas involved in multiple functions (e.g., visual or motor cortex). Thus, it cannot be concluded with absolute certainty that identified structures are involved exclusively with visuospatial function. Despite this limitation, this task provides useful information in identifying eloquent visuospatial cortical and subcortical regions to be preserved during tumor resection. Additional testing with a larger cohort would be useful in confirming the positive results of this study. 

## Conclusions

Techniques for mapping cortex involved in higher functions are continually being developed. We present the feasibility of using a target cancellation technique in awake surgery to avoid neglect postoperatively. In patients unable to complete the line bisection test, target cancellation may offer an alternative method of visuospatial mapping.
